# How does an integrated primary care approach for patients in deprived neighbourhoods impact utilization patterns? An explorative study

**DOI:** 10.1186/s12889-016-3246-z

**Published:** 2016-07-11

**Authors:** Dionne S. Kringos, Jennifer R. van den Broeke, Arnold P. M. van der Lee, Thomas Plochg, Karien Stronks

**Affiliations:** Department of Public Health, Academic Medical Center (AMC), University of Amsterdam, PO-box 22660, Amsterdam, 1100 DD The Netherlands; Kenniscentrum, Zilveren Kruis Achmea, Storkstraat 12, Leusden, 3833 LB The Netherlands

**Keywords:** Integrated delivery of health care, General practice, Use of GP care, Use of hospital care

## Abstract

**Background:**

To explore changes in utilization patterns for general practice (GP) and hospital care of people living in deprived neighbourhoods when primary care providers work in a more coherent and coordinated manner by applying an integrated approach.

**Methods:**

We compared expected (based on consumption patterns of a health insurers’ total population) and actual utilization patterns in a deprived Dutch intervention district in the city of Utrecht (Overvecht) with control districts 1 (Noordwest) and 2 (Kanaleneiland) over the period 2006–2011, when an integrated care approach was increasingly provided in the intervention district. Standardized insurance claims data were used to indicate use of GP care and hospital care.

**Results:**

Our findings revealed that the utilization of total GP care increased more in the intervention district than in the control districts. And that the intervention district showed a more pronounced decreasing trend in total hospital use as compared to what was expected, in particular from 2008 onwards. In addition, we observed a change in type of GP care use in the intervention district in particular: the number of regular consultations, long consultations, GP home visits and evening, night and weekend consultations were increasingly higher than expected. The intervention district also showed the largest decrease between actual and expected use of ambulatory care, clinical care and 1-day hospitalizations.

**Conclusions:**

Utilization patterns for general practice and hospital care of people living in deprived districts may change when primary care professionals work in a more coherent and coordinated manner by applying a more ‘comprehensive’ integrated care approach. Results support the expectation that a comprehensive integrated care approach might eventually contribute to the future sustainability of healthcare systems.

**Electronic supplementary material:**

The online version of this article (doi:10.1186/s12889-016-3246-z) contains supplementary material, which is available to authorized users.

## Background

People living in deprived neighbourhoods have higher morbidity and mortality rates [1, 2], and they become chronically ill twice as often compared to more advantaged populations [3]. They not only suffer from multimorbidity 10 to 15 years earlier in their course of life [4], they also experience relatively more complexities in other parts of their lives such as work, living conditions, income and upbringing [5].

The available evidence suggests that people in lower socioeconomic groups compared to higher groups receive more inappropriate care as regards their (often multiple) health and social care needs [6–9]. This mismatch results in unnecessary high health care consumption rates. Health care systems are not yet sufficiently geared towards treating patients with multiple health and social needs. Health care and social care services are fragmented and the provision of public health services is hardly structurally embedded in regular care [6]. In addition, many public health measures and health care approaches of professionals do not seem to fit the accumulation of health and social problems presented by patients (incl. e.g. their cultural diversity) from deprived neighbourhoods in their practice [7]. As a result, health problems remain unresolved leading to a high amount of frequent attenders in primary care [9], high referral rates from primary to specialized care, and inappropriate medication use [10–12].

The provision of integrated care is considered an important strategy for improving the quality of health care services delivery for patients with multiple care needs and reducing health care expenditures [10]. Integrated patient care implies an ability of health care professionals to “coordinate care across professionals, facilities, and support systems; continuous over time and between visits; tailored to the patients’ needs and preferences; and based on shared responsibility between patient and caregivers for optimizing health” [13]. Integrated care approaches emphasize particularly the re-organization of processes, structures and systems to enable better coordinated care geared to the need of patients [14]. This requires a close collaboration between public health, primary care and social care [6, 15–17].

Although there are examples of implemented integrated care arrangements, the evidence base on the effectiveness of integrated care remains inconclusive thus far [16, 18–20]. Amongst others, one reason is that integrated care arrangements focus on the optimizing of working processes and structures in the first place, and pay less attention to the health and social care professionals’ expertise and attitude. This additional focus is important, as the success of integrating hinges not only on organizational structures, but also on the individuals’ expertise and attitude to help patients with multimorbidity living in deprived circumstances [4, 21–24].

This theory was put into practice recently in a deprived district in The Netherlands, called Overvecht. An intervention was implemented with the aim to stimulate primary care professionals to improve their expertise, attitude, behaviour and tools to better support them to provide more comprehensive forms of integrated care. Within this area-based programme improvement of integrated care approaches led to professionals taking on a population health orientation, a focus on prevention, applying a generalist view to patients enabling the undertaking of cross domain actions, and coaching behaviour to empower patients to become participants in their own care processes and self-manage their own health [25].

Despite the high expectations of integrated care, it is currently unknown what health care utilisation patterns emerge when care is organised in a more coherent and coordinated fashion provided by competent professionals. Available research in this area is often limited to specific conditions and performed at individual patient level. Projects that have implemented such an integrated approach in a specific neighbourhood can be approached as natural experiments to study the way health care patterns evolve. Insight in appropriate health care utilization patterns of patients will support health services capacity planning, resource allocation, and will inform decision makers’ considerations when contemplating scaling up integrated care transformations.

This study therefore aims to contribute to the evidence base by exploring the primary care and secondary care utilization patterns of residents of a deprived district in The Netherlands (Overvecht) where health and social care professionals in the period 2006–2011 increasingly have renewed their expertise, attitude, behaviour and tools to provide more tailored integrated services for their citizens, as part of an area-based programme [25]. In addition, we will compare these patterns with those in two control districts.

The area-based programme was initiated by a major Dutch health insurer (Agis Health Insurance) and the municipality of Utrecht in 2006. The background of this endeavour was rooted in the high health care consumption rates, the extreme bad self-perceived health status of residents, the high morbidity, and high unemployment rate. More than 50 % of residents had at least one chronic disease, around half of residents had an increased risk of depression or anxiety disorder. In addition, one in five residents of the dictrict Overvecht were obese, half of residents were overweight, and one in three residents were socially isolated. Van den Broeke et al. (2015) [25] described the area-based programme aimed at promoting integrated care that health insurer ‘Agis Health Insurance’ with the Municipality of Utrecht implemented in the district Overvecht. Additional file 1: Table S1 summarizes the activities within the programme.

The implemented activities targeted three problems: the difficulty for professionals grasping the complexity of problems presented by patients in their practices, the difficulty with activating clients to self-manage their own health, and the fragmentation in health care provision. The interwoven problems were tackled with a more comprehensive solution consisting of ‘generalism’, ‘coaching’ and ‘population health orientation’. All primary health care professionals working in the pilot district learned how to consider patients in their social contexts (taking a more holistic view) and understand what different problems a patient might have and how these might interact. The professionals became more able to coach patients to actively take part in solving their health and social problems. They gradually succeeded in developing this comprehensive integrated care approach together and spread it throughout the district which is still an ongoing process [25].

## Methods

### Study period

The study period ranges from 2006 to 2011, the period in which the pilot interventions were implemented (Additional file 1: Table S1). A previous study has shown that during the intervention period, primary care professionals in the intervention district increasingly accustomed to a more integrated working approach over this period of time [25]. We therefore expect the impact of the integrated care approach on health care use to become increasingly stronger over the course of time.

### Study design and study population

#### Intervention district

The intervention district was Overvecht, a deprived district in Utrecht, The Netherlands. About 2/3 of all residents in Overvecht is insured at ‘Agis Health Insurance Company’, which is the main health insurance company in the central part of the Netherlands, where Utrecht is located. For those inhabitants who were insured with Agis, we derived health care utilization data from the Agis Health Database (AHD) [26]. This database includes information on payments for the provision of all medical care to its insured patients, along with demographic data of insured patients. The AHD has been shown to be representative for the urbanized areas of the Netherlands [26]. We selected data from the AHD for the whole intervention period (2006–2011).

Table 1 provides an overview of key characteristics of the intervention district. In 2006, the total number of residents in the intervention district that were insured at Agis Health Insurance was almost 20,000. Over the whole study period, 829 additional residents obtained health insurance at Agis Health Insurance.

**Table 1:** Key characteristics in 2006 and 2011 of the intervention district and two control districts in comparison to the whole of Utrecht City

Socio-demographics:	Overvecht *intervention district*	Utrecht Noordwest (only Ondiep/Zuilen-Oost) *control district 1*	Kanaleneiland *Control district 2*	Utrecht City (total)
Total population	*2006*: 31,403	*2006*: 40,613	*2006*: 15,270	*2006*: 281,011
	*2011*: 31,422	*2011*: 40,862	*2011*: 15,593	*2011*: 311,405
% 65 years or older	*2006*: 17.3 %	*2006*: 11.5 %	*2006*: 9.5 %	*2006*: 10.6 %
	*2011*: 16.0 %	*2011*: 10.9 %	*2011*: 8.7 %	*2011*: 9.8 %
Non-western immigrants	*2006*: 40.4 %	*2006*: 25.4 %	*2006*: 70.5 %	*2006*: 20.8 %
	*2011*: 45.6 %	*2011*: 25.9 %	*2011*: 70.1 %	*2011*: 21.5 %
Recipients of social benefits	*2006*: 24.8 %	*2006*: 20.3 %	*2006*: 23.6 %	*2006*: 14.3 %
	*2011*: 22.7 %	*2011*: 15.9 %	*2011*: 19.9 %	*2011*: 11.6 %
Insufficient income to manage daily living activities (self-reported)	*2006*: 10.5 %	Not available	*2006*: 17.5 %	*2006*: 6.2 %
	*2011*: 12.8 %		*2011*: 16.2 %	*2011*: 6.5 %
Medium or bad health status	*2006*: −	Not available	*2006*: −	*2006*: −
	*2011*: 25.4 %		*2011*: 26.6 %	*2011*: 13.9 %
Health care facilities:				
General practitioners per 10,000 residents	*2006*: 6.7	*2006*: *3.8*	*2006*: *4.6*	*2006*: *7.7*
	*2011*: 5.7	*2011*: 5.9	*2011*: 2.8	*2011*: 6.4
Number of multidisciplinary health care centers	*2006*: Not available	*2006*: Not available	*2006*: Not available	Not available
	*2011*: 5	*2011*: 1	*2011*: 2	
Distance to nearest hospital				
- Diakonessenhuis	- 7.0 km	- 5,1 km	- 4,2 km	Not available
Health Insurance				
Number of residents with a health insurance at Agis Zorgverzekeringen by year	*2006*: 19,291	Total in Utrecht Noordwest:	*2006*: 11,241	Not available
	*2011*: 20,120	*2006*: 19,444 *2011*: 17,833	*2011*: 11,025	

Source: [38, 39]

#### Control districts

We compared the trends in health care use in the intervention district with the trend in two control districts in the city of Utrecht which were not part of the pilot study. These districts were similar in terms of national health policy, deprivation, percentage of the population insured with Agis Health Insurance, health insurers policy and professional standards of health care providers. Furthermore, no substantial changes occurred during the study period in the delivery of primary care in these districts. These control districts were ‘Utrecht Noordwest’, which we will call ‘control district 1’ (covering the areas ‘Ondiep’ and ‘Zuilen-Oost’), and ‘Kanaleneiland’, which we will call ‘control district 2’.

In 2006, the total number of residents insured at Agis Health Insurance was approximately 20,000 in control district 1 and 11,000 in control district 2 (Table 1). In control district 1 this number decreased over the study period with approximately 10 %. In control district 2 no substantial changes occurred.

The sociodemographic composition of the population in the control districts differed in some aspects from that in the intervention district, e.g. with regard to age. For that reason, in the statistical analyses, we controlled for a number of potential confounders at the level of both the individual residents and the district (see section on statistical analyses).

### Measures

#### Health care use

Although the intervention aimed at realizing a change in the attitude, behaviour and working approaches of primary care workers in particular [25], one would expect this also to have consequences for health care consumption at hospital level, e.g. due to changing referral behaviour of general practitioners. For that reason, we included both utilization of general practice care and hospital care, as registered in the AHD, as an outcome measure.

With regard to consumption of GP care, we made a distinction between different types of care. To compute an overall consumption profile we used weighting factors that take into account the content, duration and costs of the services, as developed by the Dutch Healthcare Authority [27]. The types (and corresponding weights) are: regular consultations (1), telephone consultations (0.5), long consultations (2), evening/night or weekend consultations (2), and home visits (2).

With regard to hospital services, the AHD allows for a distinction between ambulatory care, clinical care and 1-day hospitalisation. Diagnostic/treatment codes are used for the reimbursement of hospital services. To compute an overall consumption profile for each of these hospital services, we used weight as developed by Agis Health Insurance, again taking into account the content, duration and costs of the services. The total hospital care utilization profile thus included the number (with corresponding weight) of ambulatory diagnosis/treatment codes (1), clinical diagnosis/treatment codes (10), and 1-day hospitalizations diagnosis/treatment codes (4).

#### Confounders

Several covariates were used to control for potential confounding: age (5 years categories), gender, and comorbidity (based on payments as registered in AHD for the provision of medical care in relation to a number of specified chronic conditions) at the individual level; and socioeconomic status at the level of the district (based on average disposable income, percentage social benefit recipients, percentage of residents from non-western origin, and number of residents per square kilometre).

### Statistical analyses

We assessed the trend in health care use during the intervention period (2006–2011) in the intervention district, and compared this with the corresponding trend in the control districts. Results are presented as the number of consultations for a specific service per 1000 insured residents per year. In view of the exploratory aim of the analysis, we did not test for statistical significance.

As some sociodemographic attributes differ between the intervention and control districts as well as over time (Table 1), we could not simply rely on the data on actual use of health care. To account for the sociodemographic differences, we calculated the expected use of care, controlling for the aforementioned confounders (age, sex, socioeconomic status and comorbidity) using logistic regression analysis. The total population of Agis was used as the reference group and the correction was carried out per year. In this way the changes over time in national health care policy and the policy of the health care insurer Agis are accounted for. The result is that for every person in the study population expected values based on the consumption of all Agis insures are calculated.

We will present the average number of consultations per 1000 insured residents and compare these with the number of expected consultations, for both the intervention district and control districts. In order to assess the trend over time, we will present the change in actual versus expected number of consultations using the year 2006 as index.

## Results

### Trends in total general practice care utilization and total hospital care utilization

**Fig. 1:**
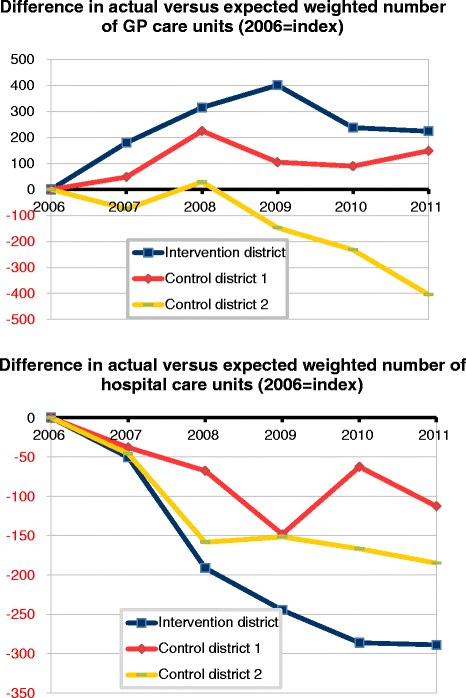
Trend in actual versus expected weighted number units, for total GP care and total hospital care, 2006–2011 (index:2006), in intervention district and control districts. **a**. Difference in actual versus expected weighted number of GP care units (2006 = index). **b** Difference in actual versus expected weighted number of hospital care units (2006 = index)

Table 2 shows the actual and expected average health care use per 1000 insured clients, regarding total GP care and total hospital care. Residents in the intervention district are using more GP care than expected from 2007 onwards. Also in both control districts, during the whole study period, the use of GP care was higher than expected. In control district 2 the number of actual versus expected total GP care decreased over time. If we relate the use of care from 2007 onwards to the index year 2006, it becomes clear that the total GP care increased more in the intervention district than in the control districts, particularly compared to control district 2 (Fig. 1).

**Table 2:** Actual and expected use of total GP care and hospital care, in intervention district and control districts, 2006-2011

Year	Intervention district			Control district 1			Control district 2		
	Number actual per 1000	Number expected per 1000	Difference actual and expected	Number actual per 1000	Number expected per 1000	Difference actual and expected	Number actual per 1000	Number expected per 1000	Difference actual and expected
average weighted number of GP care units									
2006	4.122	4.279	−157	4.135	4.128	7	4.468	4.032	436
2007	4.532	4.510	22	4.380	4.326	55	4.604	4.243	361
2008	4.763	4.605	157	4.701	4.469	232	4.849	4.384	465
2009	5.119	4.876	243	4.862	4.751	112	4.944	4.655	289
2010	5.053	4.973	80	4.974	4.878	96	4.946	4.743	204
2011	5.257	5.191	66	5.244	5.088	156	4.996	4.964	32
average weighted number of hospital care units									
2006	2.689	2.538	151	2.612	2.518	94	2.556	2.350	206
2007	2.668	2.567	100	2.637	2.581	56	2.598	2.437	160
2008	2.538	2.578	−40	2.609	2.582	26	2.488	2.441	48
2009	2.735	2.829	−94	2.864	2.918	−55	2.766	2.712	55
2010	2.858	2.994	−136	3.109	3.077	31	2.851	2.812	39
2011	2.947	3.085	−138	3.163	3.182	−19	2.982	2.960	21

In the intervention district, from 2008 onwards, there has been a decrease in total hospital care use in relation to the number expected (Table 2). Although in some years, control district 1 shows a decrease in total hospital care use, we did not observe a systematic trend. As a result, the decreasing trend in total hospital use as compared to what was expected, was more pronounced in the intervention district, in particular from 2008 onwards (Fig. 1).

### Trend in general practice use by consultation type

**Fig. 2:**
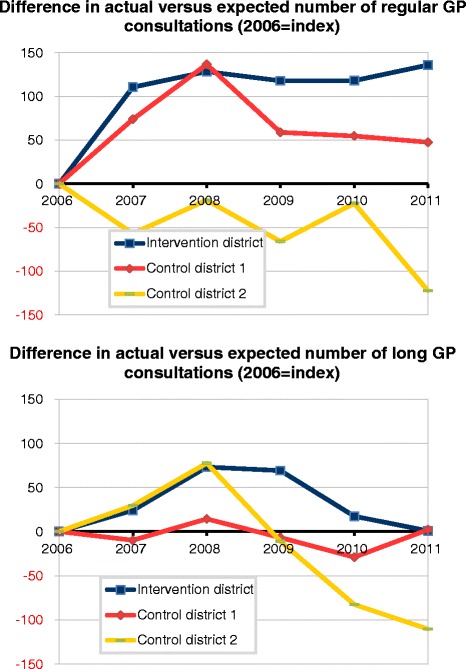
Trend in actual versus expected weighted number units, for different types of GP care, 2006–2011 (index:2006), in intervention district and control districts. **a** Difference in actual versus expected number of regular GP consultations (2006 = index). **b** Difference in actual versus expected number of long GP consultations (2006 = index)

The divergent trends in total GP care between the intervention district and the control districts appears to conceal different patterns for different types of GP use (Table 3). Regarding regular GP consultations, only residents in the intervention district had less consultations than expected (Table 3). The usage increased since 2007 but remained lower than expected during the study period. Compared to the control districts, the intervention district showed a higher increase in actual use versus expected use with regard to regular and long consultations, GP home visits and evening, night and weekend consultations. This was particularly so in comparison with district 2. As a result, for these types of GP care, we found divergent trends in the difference between actual and expected for the intervention district and control districts (Fig. 2). There were no noticeable differences between districts regarding telephone consultation (Table 3).

**Table 3:** Actual and expected use of different types of GP care, in intervention district and control districts, 2006-2011

Year	Intervention district			Control district 1			Control district 2		
	Number actual per 1000	Number expected per 1000	Difference actual and expected	Number actual per 1000	Number expected per 1000	Difference actual and expected	Number actual per 1000	Number expected per 1000	Difference actual and expected
Regular GP consultations									
2006	2.323	2.584	−262	2.566	2.511	55	2.744	2.465	279
2007	2.448	2.600	−151	2.651	2.522	129	2.698	2.476	222
2008	2.497	2.630	−133	2.745	2.554	192	2.775	2.515	260
2009	2.556	2.700	−144	2.733	2.619	114	2.799	2.586	213
2010	2.477	2.620	−144	2.667	2.557	110	2.766	2.510	256
2011	2.530	2.656	−126	2.711	2.609	103	2.707	2.550	156
Long GP consultations									
2006	246	237	9	194	234	−40	252	218	34
2007	363	330	33	267	317	−50	365	302	63
2008	450	367	82	335	360	−26	452	341	111
2009	503	425	78	369	416	−47	418	395	23
2010	521	495	27	409	477	−69	409	458	−49
2011	575	565	10	503	541	−38	447	524	−77
Evening, night and weekend GP consultations									
2006	273	247	27	261	239	22	253	247	6
2007	297	258	40	266	247	20	257	257	1
2008	278	247	32	261	242	19	248	251	−3
2009	325	269	56	295	264	31	262	273	−11
2010	292	256	36	284	253	31	250	257	−7
2011	309	268	41	292	264	29	259	269	−10
Telephone GP consultations									
2006	825	642	183	696	648	48	789	602	187
2007	874	716	158	723	715	8	771	670	101
2008	945	786	159	854	796	58	854	748	106
2009	1.070	881	189	968	912	56	989	849	140
2010	1.252	1.086	166	1.217	1.141	77	1.194	1.046	148
2011	1.296	1.131	165	1.300	1.175	125	1.247	1.092	155
Home GP visits									
2006	175	204	−29	155	173	−18	160	168	−8
2007	163	189	−25	151	160	−9	138	157	−19
2008	169	177	−8	169	157	12	125	156	−32
2009	186	173	12	159	159	0	145	154	−9
2010	162	154	8	157	145	12	133	140	−7
2011	155	152	4	146	141	4	126	141	−15

### Trends in hospital care utilization by type of service

**Fig. 3:**
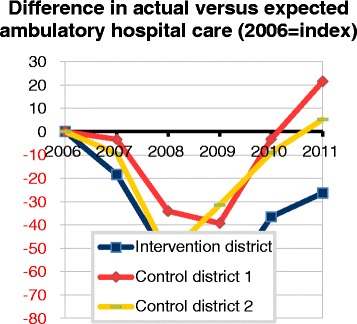
Trend in actual versus expected weighted number units, for ambulatory hospital care, 2006–2011 (index:2006), in intervention district and control districts. Difference in actual versus expected ambulatory hospital care (2006 = index)

All districts show an increase in ambulatory care from 2009 and onwards, but this remains clearly lower than expected in the intervention district, in all years (Table 4). In control district 1 ambulatory care use is only above the expected level in 2011, while in control district 2 it is well above the expected level during the whole study period. As a result, the difference between the actual and expected levels of the intervention district and the two districts increased over time (Fig. 3). Looking at clinical care, all districts show a reduction in the difference between actual and expected average use, though the numbers are small and do not seem to differ much across districts (Table 4). While the differences with the control districts are small, the intervention district shows the largest negative difference between actual and expected use of clinical care from 2009 and onwards. The number of 1-day hospitalizations of residents in all districts is above expectation, with small differences between districts. The intervention district shows the largest decrease between actual and expected use of 1-day hospitalizations.

**Table 4:** Actual and expected use of different types of hospital care, in intervention district and control districts, 2006-2011

Year	Intervention district			Control district 1			Control district 2		
	Number actual per 1000	Number expected per 1000	Difference actual and expected	Number actual per 1000	Number expected per 1000	Difference actual and expected	Number actual per 1000	Number expected per 1000	Difference actual and expected
Ambulatory care									
2006	940	952	−12	927	935	−8	960	892	68
2007	930	960	−30	937	949	−12	962	903	58
2008	805	872	−67	831	874	−42	845	830	15
2009	802	883	−81	847	894	−48	878	842	36
2010	863	911	−48	914	925	−12	918	859	59
2011	913	951	−38	972	959	13	971	898	73
Clinical care									
2006	143	132	11	137	131	7	132	121	11
2007	138	132	7	135	133	2	133	126	7
2008	140	140	0	140	138	3	132	132	−0
2009	154	161	−6	159	164	−5	151	154	−3
2010	160	171	−11	171	174	−2	153	159	−6
2011	161	173	−12	170	177	−7	160	167	−7
1-day Hospitalizations									
2006	80	67	13	78	69	9	69	63	6
2007	88	72	16	88	77	11	76	68	8
2008	82	76	6	93	83	10	81	73	9
2009	98	85	13	106	95	11	93	82	12
2010	100	94	6	121	104	17	100	90	10
2011	105	101	5	123	113	10	103	97	6

## Discussion

### Main findings

Our study found changes in utilization patterns for general practice and hospital care of people living in deprived districts when primary care professionals work in a more coherent and coordinated manner by applying a comprehensive integrated care approach. We compared utilization patterns in the intervention district with those in two control districts over the period 2006–2011, while an integrated care approach was increasingly provided in the intervention district.

Our findings revealed that the utilization of total GP care increased more in the intervention district than in the control districts. And that the intervention district showed a more pronounced decreasing trend in total hospital use as compared to what was expected, in particular from 2008 onwards. In addition, we observed a change in type of GP care use in the intervention district in particular: the number of regular consultations, long consultations, GP home visits and evening, night and weekend consultations were increasingly higher than expected. The intervention district also showed the largest decrease between actual and expected use of ambulatory care, clinical care and 1-day hospitalizations.

### Explanation of results

The observed changes in the utilisation patterns in the intervention district are timely and relevant. Overvecht mirrors the initial dynamics within Dutch primary care featuring the development of more comprehensive integrated care arrangements to include professional competencies and attitudes as well [21, 25, 27]. The intervention has drawn much attention in The Netherlands [28], and its underlying principles are reinforced by the white paper of a national advisory committee on innovating healthcare professions and education [29]. At the international level it voices the call for stronger primary care systems enabling a more generic comprehensive approach towards multimorbidity [4, 30].

In this perspective, our study findings supports the further exploration of this comprehensive integrated primary care, linking it up to the debates on reconfiguring health professionalism [21], on the modernising of education and new professional expertise [31], on the self-management by patients [32], on integrating public health and primary care [5, 15–18], and on applying complex adaptive systems thinking within primary care [33, 34].

Above all, the key contribution of our study lies in the dynamics in health care use found. Our study suggests that substituting GP care for hospital care is possible. Many health policies across countries aim to achieve this substitution, while supporting evidence is limited [35, 36].

It further may shed some light on the way more comprehensive primary care could absorb hospital use. Our study shows that GP’s do spend more time on patients with an accumulation of problems, resulting in an increase in the number of regular 10 min and longer consultations. This goes along with GP’s seeing more patients during out-of-office hours, which may reflect an improved accessibility and continuity of GP care, potentially reducing unnecessary Emergency Room visits as well. This result is in accordance with the findings of a recent international survey in 34 countries [37].

In addition to the above mentioned explanations related to the behaviour of the provider, the impact on health care use might also be the result of changing health behaviour of patients. Unfortunately, no data are available to further examine this factor. It is recommended that future research tries to disentangle the effects of the interventions directed at providers and at patients.

### Strengths and limitations of this study

Our study provides a unique exploration of the potential impact a comprehensive integrated care approach could have on health care use for people in deprived districts. Its strength lies in the large number of respondents included in the study and the two control districts. Moreover, the Agis Health Insurance sample included almost 2/3 of the residents in Overvecht. In previous studies, this population has been proven to be representative for the total Dutch urban population [26], limiting the risk of selection bias. Given the gradual uptake of the integrated care approach from 2006 and onwards [25], we could only warrant the comparability of data by including data from 2006 to 2011 for which it was also likely that an increasing impact in health care use would have occurred. Before 2006 and from 2012 onwards, data were incomparable as the financing system of healthcare in The Netherlands changed. Ideally, we would have included utilization data for other primary care providers, medication use, and social care providers, but this was not possible for reasons of data comparability, availability and complexities such as the introduction of new professionals over time. We therefore recommend further research in studying the impact on other primary care services, public health, and social care services.

The somewhat similar direction of trends in the intervention district and control district 1 (although they differed in their intensity of change) may point to spill over effects potentially caused by the media attention for the intervention. If this is the case, this means our findings in the intervention district are an underestimation of the actual impact of the integrated care approach on health care use.

Finally, we have checked the stability in composition of insured clients over time, and only noticed small changes. We do not expect this to have influenced the changes in health care utilization we found because we used standardized utilization data, and because the differences between districts remain constant over time.

## Conclusions

Utilization patterns for general practice and hospital care of people living in deprived districts may change when primary care professionals work in a more coherent and coordinated manner by applying a more ‘comprehensive’ integrated care approach. Results supports the further exploration of the potential of ‘comprehensive’ integrated care embedded in primary care in deprived neighbourhoods to the future sustainability of healthcare systems.

## Additional file

Additional file 1: Table S1. Activities implemented in Utrecht Overvecht to promote renewal of expertise from 2006-2011. (DOCX 19 kb)
