# Supplementary material for: How does an integrated primary care approach for patients in deprived neighbourhoods impact utilization patterns? An explorative study
Source: BMC Public Health. 2016 Jul 11;16:545. doi: 10.1186/s12889-016-3246-z (PMC4940836; doi:10.1186/s12889-016-3246-z)
Supplement: Additional file 1: Table S1. — Activities implemented in Utrecht Overvecht to promote renewal of expertise from 2006-2011. (DOCX 19 kb) [file 12889_2016_3246_MOESM1_ESM.docx]

| **Additional file 1: Table S1** Activities implemented in Utrecht Overvecht to promote renewal of expertise from  2006-2011 | |
| --- | --- |
| **Year** | **Activity** |
| 2006 | - Implementation of the Big!move foundation (B!M) by one of the multidisciplinary healthcare centers (MHCC). Big!Move is a programme for exercise based on the viewpoint that the focus should be shifted from illness and cure (I&C) to health and healthy behavior (H&H), (I&C🡪H&H). This programme or method was developed in Amsterdam South-east (Aalders et al. 2010). The principle is to challenge patients not to show their dependence, but to empower themselves with doing the things they are able to do - Continuation of GO, a joint initiative to prevent overweight and obesity aimed at children and their parents started in 2005 by the municipality Utrecht, the Welfare Service, and the major Homecare Service in Overvecht, and coordinated by the department of Public Health (PH dept.) (De Geus et al. 2011) |
| 2007 | - Within the B!M stronger emphasis is put on the organizational development of primary care and the further development of the programme for exercise - Start Happinezz, a programme for professionals providing consultation for complex cases. Through intervision [?intervisie] colleagues form different professionals give feedback to help improve a professionals’ care or support to a certain patient. Parties involved in the development of Happinezz: B!M, several MHCC ,Welfare Service, Homecare Service, Mental healthcare Service, psychologist and ‘Cliëntenbelang Utrecht’ (a patient organization for all patients in the municipality of Utrecht) |
| 2008 | - Start El Kouaa, an empowerment project for Moroccan women. Parties involved in the development of El Kouaa: B!M, several MHCC, Welfare Service, Mental healthcare Service - Further development of Happinezz - Further developing B!M - May: first round of dialogue in Overvecht between the major healthcare insurance company Agis Zorgverzekeringen (HC.Ins.Comp.), the PH dept. and health and social care professionals from local organizations - October: Signing of the memorandum of understanding on the 8^th^ of October by HC.Ins.Comp. and the municipality of Utrecht - December: a first version of the Plan of action is written by the project leaders from the HC.Ins.Comp. and the PH dept. in collaboration with the first group of involved professionals from local organizations, building upon the earlier initiatives Happinezz and El Kouaa |
| 2009 | - February: start Project Group Healthy Neighborhood Overvecht (HNO). Parties involved: the PH dept., HC.Ins.Comp., Welfare Service, Mental healthcare Service, three MHCC and B!M - First steps are taken to establish collaboration between three MHCC to achieve integrated primary care, which will later become the ‘Overvecht GEZond!’ foundation (OG!). Involved are three MHCC and B!M. - June: To warrant the activities developed within the Project Group HNO at management- and policy level, a Director Group is initiated in which the directors of the Welfare Service, Mental Healthcare Service, the major Homecare Service, OG!, HC.Ins.Comp. and PH dept. take place. The Project Group becomes a Platform. Middle 2009 the Platform HNO and the Director Group HNO are functioning at full strength. - The Platform HNO starts the project ‘One language’. An independent advisor is asked to perform the project to take stock of and analyse the problems professionals in Overvecht experience and possible solutions - Further development of Happinezz - The Platform HNO starts the Project Vitamin D. A campaign for awareness of the consequences of lack of vitamin D, as an experiment in collaboration between PH and PC professionals - The Platform HNO starts the project ‘Exercise broker’, a project to encourage adults and the elderly to exercise more - OG! does a grant application with the support of the PH dept. and the HC.Ins.Comp. The grant is for ‘Zichtbare Schakel’ (‘ZS’) a subsidy by the national government to encourage the use of district nurses - OG! does a grant application with the support of the PH dept. and the HC.Ins.Comp. The grant is for ‘SOLK’ a subsidy by the national government to encourage integrated care for patients that have complaints that cannot be explained by a physical component |
| 2010 | - OG! becomes an official foundation and a fourth MHCC joins OG! - The Platform HNO and Director Group HNO organize the First Neighborhood Conference in which the HNO viewpoint (based on I&C🡪H&H) is explained and tools are provided to make it possible to work by this viewpoint - Propaganda for the Happinezz method through a publication and a workshop at the First neighborhood Conference - The grant for ‘SOLK’ is honored and OG! starts the project ‘SOLK’, a project to learn how to help patients that have complaints that cannot be explained by a physical component - The Platform HNO starts the ‘Healthy Information Table’, a campaign for healthy lifestyle of the inhabitants of Overvecht - The Project Vitamin D is held a second time - The Platform HNO encourages that a lot of effort is being made to realize that more adults exercise. This is done through connecting primary care services to the Welfare Service - The grant for ‘ZS’ is honored and OG! will work together with a small Homecare Service |
| 2011 | - A fifth MHCC joins OG! and B!M becomes assimilated into the foundation - The major Homecare Service joins the Platform HNO and the Director Group HNO - The Platform HNO and Director Group HNO organize the Second Neighborhood Conference - Further development of the HNO viewpoint and the analysis of the expertise professionals need in order to work by the HNO viewpoint - Implementation of the Happinezz method in all centers that are joined in OG! - Further development of the methods developed within ‘ZS’ and ‘SOLK’ - The Platform HNO starts the Project Relax in Overvecht. A campaign about relief of stress aimed at inhabitants with mild psychological complaints - Continuation of the activities aimed at exercise - Development of a project to consult a physiotherapist for exercise based on the viewpoint of I&C🡪H&H |
| 2012 | - The Platform HNO and Director Group HNO organize the Third Neighborhood Conference - The HNO viewpoint and expertise are recorded - The hospital joins the Platform HNO and the Director Group HNO - A new multidisciplinary team ‘Neighborhood team Strong Overvecht’ that supports adults and families with multiple complex health and social problems joins the Platform HNO and the Director Group HNO - The start of a collaboration with ‘Strong Basic Care’(connecting medical-social) - Further development of the methods developed within ‘ZS’ and ‘SOLK’ - Continuation of the activities aimed at relief of stress with The Month of Relaxation - A logo and slogan for HNO are developed, a first newsletter is sent to all professionals that signed up for it, and a LinkedIn group was created - The Platform HNO starts the Gaming consisting of several projects such as ‘Walk around the block’ and ‘Virtual Patient’ - Because of financing problems the exercise programme B!M has to stop. All other activities still go through |
| Source: | (27) |
|  | |
